# Adeno-associated-virus-mediated transduction of the mammary gland enables sustained production of recombinant proteins in milk

**DOI:** 10.1038/srep15115

**Published:** 2015-10-14

**Authors:** Stefan Wagner, Rosemary Thresher, Ross Bland, Götz Laible

**Affiliations:** 1 AgResearch, Ruakura, Hamilton, New Zealand; 2 AgResearch, Grasslands, Palmerston North, New Zealand

## Abstract

Biopharming for the production of recombinant pharmaceutical proteins in the mammary gland of transgenic animals is an attractive but laborious alternative compared to mammalian cell fermentation. The disadvantage of the lengthy process of genetically modifying an entire animal could be circumvented with somatic transduction of only the mammary epithelium with recombinant, replication-defective viruses. While other viral vectors offer very limited scope for this approach, vectors based on adeno-associated virus (AAV) appear to be ideal candidates because AAV is helper-dependent, does not induce a strong immune response and has no association with disease. Here, we sought to test the suitability of recombinant AAV (rAAV) for biopharming. Using reporter genes, we showed that injected rAAV efficiently transduced mouse mammary cells. When rAAV encoding human myelin basic protein (hMBP) was injected into the mammary glands of mice and rabbits, this resulted in the expression of readily detectable protein levels of up to 0.5 g/L in the milk. Furthermore we demonstrated that production of hMBP persisted over extended periods and that protein expression could be renewed in a subsequent lactation by re-injection of rAAV into a previously injected mouse gland.

The development of genetic engineering and the explosion of fundamental knowledge about gene structures and functions make it feasible to produce large quantities of almost any desired protein in a variety of systems ranging from simple microbial systems such as bacteria and yeast to complex eukaryotic systems such as cultured mammalian cells, transgenic plants and animals. Proper functionality of mammalian proteins is often dependent on complex post-translational modifications (mainly glycosylation), which is only achievable in cultured mammalian cells and transgenic animals^1^. Although fermentation with mammalian cells is presently the dominant technology, it has significant shortcomings including the high variability of glycosylation in response to subtle changes in culture conditions^2^, high capital costs for new production facilities and the lack of scalability^3^. The high protein production capacity of the mammary gland, together with its ability to consistently perform complex post-translational modifications and provide easy access to large quantities of the produced protein in milk, renders transgenic dairy animals one of the best bioreactors for the production of high value mammalian proteins^4^.

The biopharming of transgenic animals for the production of pharmaceuticals in milk has been fully validated with the market approval of the first transgenic animal-derived biopharmaceutical, the recombinant human antithrombin ATryn, in Europe^5^ and the USA^6^ and, more recently, the commercialization of Ruconest, an esterase inhibitor for the treatment of dermal swellings^7^. However, the expense of generating transgenic founder animals and the time it takes until the first natural lactation occurs, remain major obstacles for this approach. An alternative strategy is to directly introduce gene constructs into the secretory epithelial cells of the mammary gland. Such somatic gene transfer can be applied directly to a lactation-competent animal with the potential to provide rapid production of a recombinant protein. Somatic gene transfer of the mammary gland has several advantages over the production of transgenic animals, such as lower development costs, speed and flexibility, while retaining the main advantages of the transgenic animal concept—high capacity, complex post-translational modifications, ease of collection and scalability.

Somatic gene transfer by *in vivo* transfection of the mammary gland with lipofection and electroporation following transductal delivery of vectors has been demonstrated but it achieves only poor levels of transient expression^8^. Somatic gene transfer using retroviruses (Gibbon ape leukemia virus/Moloney murine leukemia virus hybrid retrovirus) has also been assessed for the expression of human growth hormone in the goat mammary gland^9^. However, as retroviruses only transduce dividing cells, multiple transductal injections of the retrovirus were required and observed expression of heterologous proteins in milk was very low and transient.

Replication-defective adenoviral vectors, which, in contrast to retroviruses, do not integrate into the genome, have also been used. Successful expression of various pharmaceutical proteins by adenovirus-mediated somatic gene transfer has been reported in goats^10^,^11^,^12^,^13^,^14^,^15^,^16^,^17^, rabbits^18^,^19^,^20^ and mice^14^. The recombinant protein expression levels were comparable with production in transgenic animals which are commonly in the g/L range.

However, a major limitation of the adenoviral vector approach is that the adenoviral vectors are known to support only transient expression, lasting approximately one week. Only two studies were able to demonstrate expression of the recombinant protein for up to three weeks^11^,^21^. This poor persistency is generally believed to be caused by the induction of a potent immune response against the virus^22^. This potent immune response is furthermore disadvantageous as it prevents readministration of the transducing vector^23^. The lack in persistency is a significant limitation of the adenoviral approach and it is of particular importance when considering that the seasonal lactation period is 10 months for goats and cattle. Thus, there is a need for alternative means of mediating long-term expression of a transduced protein in these systems.

Wild-type AAV is not associated with any human or animal disease, has very low immunogenicity^24^ and has been previously used in human gene therapy in 92 clinical trials^25^. AAV is a small, non-enveloped, single-stranded DNA virus. The genome (~4.7 kb) contains two open reading frames, *rep* and *cap*, encoding the non-structural proteins necessary for replication and the structural viral capsid proteins, respectively. The open reading frames are flanked by the palindromic 145 bp inverted terminal repeats (ITRs), the only *cis*-acting viral sequence required for packaging. In recombinant AAV vectors, the *rep* and *cap* sequences are replaced with up to ~5 kb of a transgene cassette, thus recombinant vectors are replication incompetent and do not express any viral genes. For production of rAAV the *rep* and *cap* genes are supplied in *trans* along with adenoviral helper functions on separate plasmids^26^. The gene construct introduced by rAAV vectors stably persists episomally and is capable of mediating long term transgene expression in mammalian somatic cells^27^. There are over 100 different AAV serotypes^28^ that vary in the amino acid sequence of their capsid protein. Different AAV serotypes are better suited for transduction of different cell types. For example, AAV5 and AAV8 have successfully been used to transduce epithelial cells of the lung, gastrointestinal tract and retina^29^,^30^ whereas AAV9 has been successfully used to transduce lung cells^31^. However, to date no AAV serotype has been used to successfully transduce mammalian epithelial cells of the mammary gland of pregnant or lactating mammals.

In this study, we injected rAAVs encoding hMBP into the mammary gland of mice and rabbits. We show persistent hMBP secretion in the milk of injected animals and demonstrate that AAV1 and AAV9 can be used for mammary gland transductions. Renewed hMBP-secretion following a second injection in a previously injected animal suggests the absence of a significant immune response and thus the potential for readministration of rAAVs for recombinant protein production in the mammary gland.

## Results

### Transduction of the mouse mammary gland by different AAV serotypes

**Figure 1:**
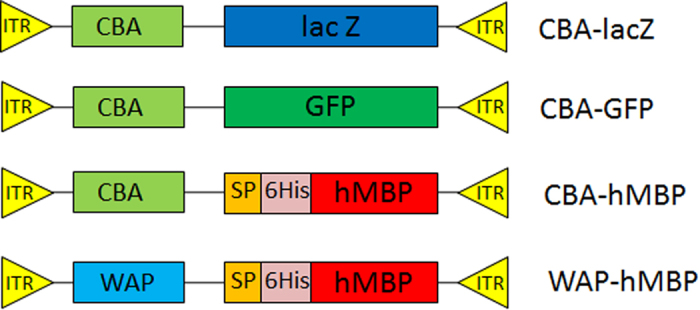
Schematic representation of the four rAAV expression constructs. The main functional elements are depicted with ITR—inverted terminal repeats, CBA—chicken beta actin promoter, WAP—mouse whey acidic protein promoter, lacZ—cDNA encoding beta-galactosidase, GFP—green fluorescent protein, SP—WAP signal peptide, 6His—six histidine tag, hMBP—cDNA encoding human myelin basic protein.

**Figure 2:**
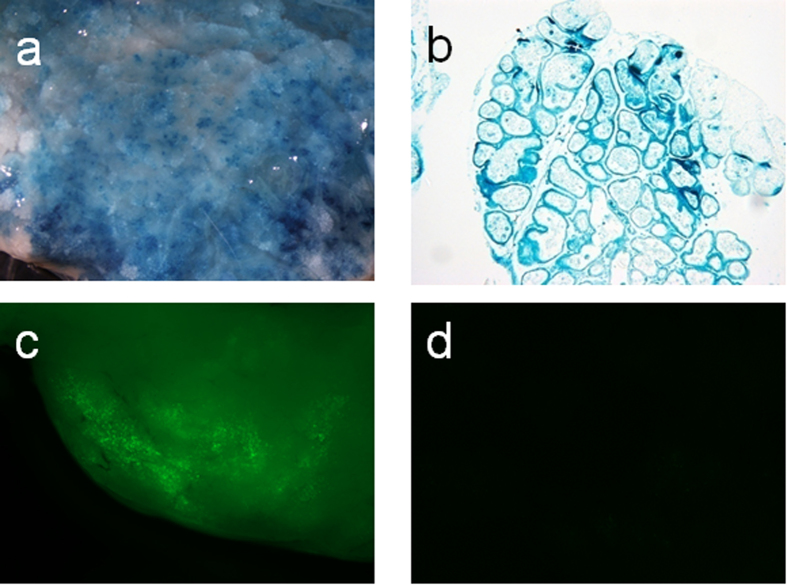
rAAV1 and 9-mediated β-galactosidase and GFP expression in murine mammary tissue. X-gal stain of mammary tissue (**a**): whole mount *in situ*, (**b**): tissue section after rAAV1 CBA-lacZ (1.3 × 10^11^ vg) injection. Visualization of GFP-expression in mammary tissue after injection of (**c**) rAAV9-CBA-GFP (2.5 × 10^10^ vg) and (**d**) AAV8-CBA-GFP (2.5 × 10^10^ vg).

AAVs consist of more than 100 slightly differing isoforms and conventional wisdoms dictates that they evolved by optimizing their ability to efficiently transduce different hosts or tissues. In our initial experiments, we choose to test whether AAV serotypes 1, 8 and 9, which are known to transduce other epithelia, were suitable for mammary tissue transduction and result in the production of recombinant proteins. First, we injected 1.3 × 10^11^ vector genomes of rAAV1-CBA-lacZ (Fig. 1) in the mammary gland of a day 18 pregnant mouse (Fig. S1). The injected mammary gland and a non-injected control gland were isolated 13 days after parturition and stained for beta-galactosidase activity with X-gal. Βoth whole mount *in situ* and tissue sections from the injected gland displayed a bright and distinctive blue stain indicative of the presence of beta-galactosidase (Fig. 2a,b) whereas non-injected control glands did not (data not shown). While we did not aim to determine the exact percentage of beta-galactosidase-positive cells in tissue from the injected gland, the whole mount *in situ* and tissue sections confirmed that a large percentage of the mammary tissue was transduced. Next, to evaluate AAV8 and AAV9, we injected 2.5 × 10^10^ vector genomes rAAV8-CBA-GFP and rAAV9-CBA-GFP in the mammary gland of a non-lactating mouse in late pregnancy that was sacrificed after eight days of lactation. Strong GFP fluorescence was observed in the injected gland that was injected with AAV9 (Fig. 2c) whereas GFP fluorescence was hardly detectable in the AAV8-injected gland (Fig. 2d).

### rAAV-mediated production of recombinant hMBP in the milk of injected mice

**Figure 3:**
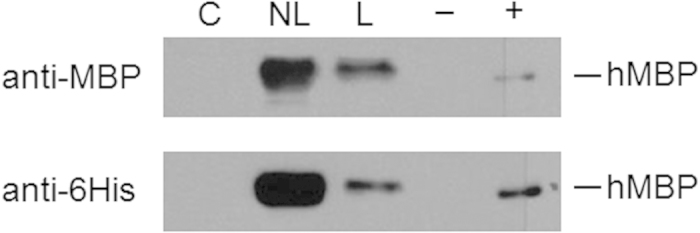
Production of hMBP in the milk of rAAV1-hMBP injected mice. Western blot detection of rhMBP in the milk of rAAV1-CBA-hMBP and rAAV1-WAP-hMBP (3 × 10^11^ vg total) injected mice. anti MBP—MBP antibody detection. anti 6His antibody—6His antibody detection. C—control, not injected. NL—non-lactating mouse, injected, L—lactating mouse, injected,— —wild type cow’s milk, + − MBP cow’s milk.

Having established that both rAAV1 and rAAV9 are capable of efficiently transducing mouse mammary tissue, we next tried to determine if mammary transduction would also result in the production and secretion of detectable levels of recombinant protein in milk. We considered using either rAAV1 or rAAV9 for these subsequent experiments, but chose to continue with AAV1 preparations since we had higher virus titers available for AAV1 preparations. We previously generated transgenic cows that secrete hMBP in their milk^32^ and decided to use this proven transgene cassette, where a WAP promoter drives expression of a 6His-hMBP fusion protein (Fig. 1). Since AAV injection in the mammary gland is likely to restrict transduction and expression of the recombinant protein to the mammary gland, we also tested a construct with a constitutive CBA promoter^33^ (CBA-hMBP) (Fig. 1). To maximize the rAAV titer for an initial pilot experiment, we used a mix of rAAV1-WAP-MBP and rAAV1-CBA-MBP with 3 × 10^11^ vector genomes for injection into the mammary gland of a non-lactating mouse at day 18 of gestation. Analysis of milk one week post parturition demonstrated hMBP expression by western blot using both MBP-specific- and His-tag-recognizing antibodies (Fig. 3).

Since it had been shown previously that the timing of adenovirus injection was crucial to achieving widespread transduction of the secretory epithelial cells^14^,^16^ , we performed comparative injections pre and post lactation. Compared to the mouse injected prior to parturition, milk produced by a mouse that was injected while lactating (day 1 past parturition) appeared to contain considerably less hMBP (Fig. 3). Therefore, to allow for optimal transduction of the mammary epithelium, all subsequent mammary gland injections were performed during the last trimester of gestation between one and five days prior to parturition.

**Figure 4:**
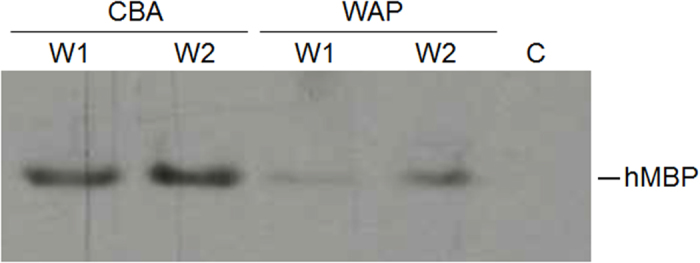
Injections of rAAV1-CBA-hMBP and rAAV1- WAP-hMBP result in secretion of MBP in milk. Western blot analysis with a MBP-specific antibody of milk samples from injected glands. CBA—milk from a rAAV1-CBA-hMBP (4.7 × 10^9^ vg) injected gland. WAP—milk from a rAAV1-WAP-hMBP (4.7 × 10^9^ vg) injected gland. C—control, milk from a non-injected gland. W1—one week post parturition, W2—two weeks post parturition.

Next, we evaluated whether CBA-hMBP and WAP-hMBP are equally efficient expression constructs for the production of hMBP in milk. Equal vector titers (4.7 × 10^9^ vector genomes) of rAAV1-CBA-hMBP and rAAV1-WAP-hMBP were injected in opposite glands of a mouse and milk from the injected glands was analyzed for the presence of hMBP. Both rAAV1-CBA-hMBP and rAAV1-WAP-hMBP triggered hMBP secretion in milk but rAAV1-CBA-hMBP injections consistently yielded more of the recombinant protein (Fig. 4). We further corroborated this finding by determining minimal titers required for hMBP detection. For rAAV1-WAP-hMBP, at least 4.7 × 10^9^ vector genomes were required to produce detectable hMBP levels in milk whereas as few as 1.7 × 10^9^ vector genomes of AAV1-CBA-MBP were still sufficient to trigger hMBP expression (data not shown).

### Sustained production of rhMBP in mice

**Figure 5:**
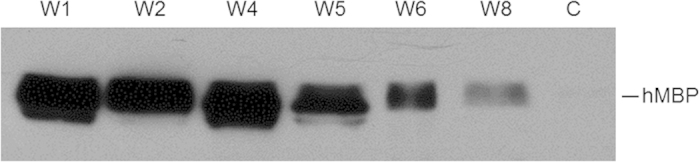
Extended hMBP expression in mouse milk. Western blot analysis with a MBP-specific antibody of milk samples from a rAAV1-CBA-MBP (2 × 10^11^ vg) injected, cross-fostered mouse. W1 to W8—one to eight weeks post parturition. C–control, milk from a non-injected gland.

Adenovirus-driven expression of a recombinant protein in mammary tissue is short-lived due to a strong systemic immune response by the host^22^. To test whether AAV-driven expression would be more sustainable, we first analyzed hMBP expression levels over the normal lactation time and later extended lactation beyond weaning of the pups by cross-fostering injected mice with younger pups. During an extended milking period of eight weeks, we observed slightly decreasing hMBP expression levels throughout the first six weeks which was followed by a considerable drop of expression in week eight (Fig. 5).

**Figure 6:**
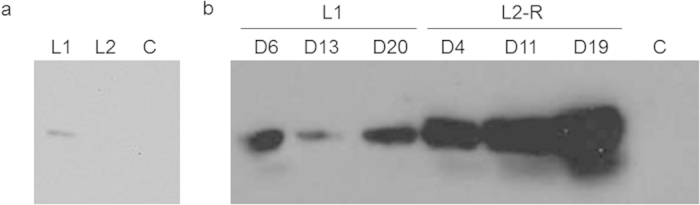
hMBP is no longer expressed in a subsequent lactation but expression can be renewed by a second injection. Western blot detection of rhMBP in first lactation (L1) and second lactation (L2) milk samples. (**a**) milk samples derived from a gland injected once with rAAV1-CBA-hMBP (1.9 × 10^10^ vg) prior to the first lactation. (**b**) milk samples from a gland injected (rAAV1-CBA-hMBP, 4.7 × 10^9^ vg) prior to the first lactation and again (rAAV1-CBA-hMBP, 8.3 × 10^10^ vg) prior to second lactation (L2-R). D—day. C—control, milk from a non-injected gland.

Given that we could still detect low levels of hMBP at the end of a prolonged lactation, we wondered whether rAAV-induced expression would persist beyond involution into a subsequent period of lactation. Analysis of milk samples one week post parturition of a mouse injected with rAAV1-CBA-hMBP showed hMBP expression during the first lactation, but we were unable to detect hMBP in the milk from the second lactation (Fig. 6a).

Recombinant protein expression in milk clearly aims at biopharming applications where it would be beneficial and desirable if the protein-producing animals could be utilized repeatedly, either for the expression of a specific protein or the sequential expression of different proteins. Since expression of hMBP does not carry over into a second lactation, we tried to determine whether we could re-establish expression by a second rAAV administration in a previously injected animal. Figure 6b shows that re-injection of higher titer rAAV1-CBA-hMBP (8.3 × 10^10^ vg compared to 4.7 × 10^9^ vg for the first injection) in the previously injected teat enables renewed hMBP production in a subsequent lactation.

### rAAV-mediated production of recombinant hMBP in rabbits

**Figure 7:**
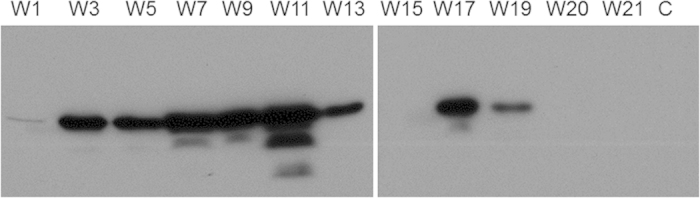
Extended hMBP expression in rabbit milk. Western blot with a MBP-specific antibody of milk samples from a rAAV1-CBA-hMBP (3.2 × 10^12^ vg) injected rabbit. W1 to W21–one to 21 weeks post parturition. C–control, milk from a non-injected gland. For clarity, a cropped image of the two gels/blots that were processed in parallel is presented. The original uncropped image is shown in Supplementary Fig. S2.

**Figure 8:**
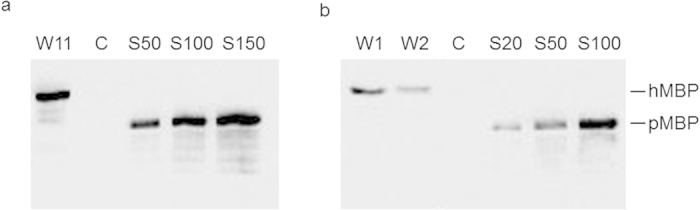
Quantification of recombinant hMBP produced in rabbit and mouse milk. Western blot analysis of MBP in milk samples from (**a**) a rabbit injected with rAAV1-CBA-hMBP (3.2 × 10^12^ vg) and (**b**) a mouse injected with of a mix of rAAV1-CBA-hMBP and rAAV1-WAP-hMBP (3 × 10^11^ vg) compared with known amounts of commercially available purified hMBP (pMBP, S20 to S150: 20 to 150 ng purified hMBP). W–week.

Biopharmaceutical exploitation of rAAV-driven protein expression relies on the manufacture of recombinant proteins in amounts unlikely to be achievable in a small animal such as the mouse. Accordingly, the two milk-derived recombinant proteins currently commercially-available are produced in goats’ and rabbits’ milk clearly supporting the notion that animals larger than mice will be needed to upscale rAAV-driven protein production. We therefore wanted to next validate the rAAV approach in rabbits which are commercially used for bio-pharming^7^. Compared to the injection in mice, we increased the virus titer more than tenfold and injected 3.2 × 10^12^ vector genomes of rAAV1-CBA-hMBP in the teat of a non-lactating, day 28 pregnant rabbit. Milk samples were taken once a week and lactation was prolonged by keeping female kids together with the injected mother for a total of 21 weeks. hMBP was detected in milk from the injected gland whereas we did not observe hMBP in milk from a non-injected gland (Fig. 7). We observed hMBP expression, albeit at varying levels, over 19 weeks of lactation. For reasons unknown, we were unable to detect hMBP in the week 15 sample. The hMBP expression levels tailed off towards the end of the analyzed lactation period and hMBP was no longer detected in week 20 and 21 milk samples (Fig. 7). To determine hMBP expression levels in rabbits, western signals for hMBP in mid lactation milk samples were quantified against known amounts of hMBP standard. The concentration of hMBP in milk of injected rabbits was determined to be approximately 0.5 g/L (Fig. 8a) which was similar to the 0.4 g/L that we observed in mice (Fig. 8b).

## Discussion

Biopharmaceutical production of recombinant proteins is a competitive multibillion dollar industry that utilizes organisms as diverse as unicellular bacteria, yeast, or algae or complex expression systems such as cultured mammalian cells, animals or plants. When choosing an expression system, two main questions have to be addressed: Does the host system yield functional protein? Is the production of the recombinant protein economical or are there cheaper alternatives? To date, most biopharmaceuticals that require complex posttranslational modifications are produced in mammalian cell culture but the production and commercialization of recombinant proteins from milk of genetically-modified animals has highlighted the potential for this less-costly *in vivo* approach; however, restricting the genetic modification to only the protein-producing organ would be cheaper, simpler and faster. We extended the concept of transducing the mammary epithelium with recombinant adenovirus, to test rAAV efficacy for the production of recombinant proteins in the mammary gland. Intriguingly, the main results of our study show that the rAAV approach compares favourably to adenovirus in various ways. Although higher initial expression levels of up to 4.8 g/L (compared to 0.5 g/L with our rAAV approach, albeit with only moderate virus titers) have been reported for the adenovirus-driven expression of some recombinant proteins, e.g. human antithrombin^20^, importantly, we did not observe the dramatic drop in protein production that all adenoviral expression studies have been hampered with^10^,^11^,^12^,^13^,^14^,^15^,^16^,^17^,^18^,^19^,^20^,^34^. Adenoviral-driven protein expression in the mammary gland typically peaks within the first week and then drops to levels that are no longer or barely detectable. In contrast, rAAV induces fairly persistent protein expression over normal lactation periods of three weeks in mice and three to five weeks in rabbits^35^. Remarkably, the production of the recombinant protein extended beyond those periods as we were able to detect hMBP for up to 8 weeks in mice and 19 weeks in rabbits with artificial extension of the lactation period. hMBP expression levels decreased at the end of lactation both in mice and rabbits which is concordant with a model postulating that the rapid cellular turnover in lactating mammary epithelium dilutes the replication-deficient rAAV. In mice, we were initially still able to detect residual amounts of the recombinant protein at the end of a prolonged lactation but this weak expression did not carry over into a second lactation. Since involution is characterized by widespread apoptosis resulting in the death of most epithelial cells, we hypothesize that rAAV is either completely lost after involution or its residual remainders, possibly surviving in mammary epithelial stem cells, no longer support the expression of detectable levels of hMBP. rAAV did, however, allow at least a second round of injection-triggered protein expression whereas a strong cellular immune response against the adenoviral capsid protein^22^ prevents re-stimulation of recombinant protein production with adenovirus-administrations. Thus, rAAV injections can be repeated in the same animal which allows “recycling” of animals. Our results also suggest that potentially animals can be used for the production of different proteins in successive lactations by simply injecting rAAV for a different protein. This would allow for rapid adjustments of production capacities according to changing market demands.

How do virus copy numbers correlate with the expression of the recombinant protein and how does AAV compare with adenovirus? As one would expect, rAAV-driven expression of recombinant proteins is dependent on a minimal virus titer. Dipping below the threshold of a certain virus titer abrogates production of detectable amounts of the recombinant protein (observed for mouse mammary gland injections with rAAV1-WAP-hMBP when compared with injections of rAAV1-CBA-hMBP). In our experience, increased virus titers correlated with increased amounts of hMBP though we do not believe we exhausted the production potential with the titers we utilised in this study.

How much protein a given recombinant virus yields may also be dependent on the choice of the promoter as we observed relatively weak expression of hMBP with rAAV1 WAP-hMBP while equal amounts of rAAV1-CBA-hMBP induced stronger expression of hMBP. This difference in expression is likely due to different promoter strength: WAP is only a minor milk protein and driven by a comparatively weak, lactation-specific promoter whereas CBA is a strong constitutive promoter optimised for high recombinant protein expression^33^.

We observed expression levels of 0.5 and 0.4 g/L in rabbits and mice, respectively which is within the 0.35 to 4.8 g/L range achieved by adenoviral transductions of rabbits and mice^14^,^18^,^19^,^20^,^34^. It is also comparable to the average production levels that have been achieved with transgenic rabbits. Dependent on the specific promoter and gene of interest combination, expression levels can be highly variable and range between 0.3 μg/L for a large, complex protein and 8 g/L for a bovine milk protein^36^. The approved human drug ATryn, a recombinant human antithrombin, is produced in the milk of transgenic goats at 2 to 3 g/L^37^ and similar to the milk expression levels we detected for hMBP in a transgenic cow model^38^. Thus, the rAAV-mediated hMBP expression levels of 0.5 g/L in our study are well within the range of adenoviral and transgenic approaches and only slightly lower compared to the hMBP production levels in transgenic cows. Considering that there still is immense scope for optimization of experimental parameters such as viral titers, volumes injected, assaying other time points for injections or screening other serotypes or AAV mutants^39^ to increase the transduction efficiency, the mammary gland rAAV-injection approach provides a very promising alternative to transgenic animals. Notwithstanding further optimization, even with the expression levels that we obtained, the AAV injection approach could be readily used for proteins with sufficiently high values or simply to test different expression constructs/variants, enable the establishment of purification schemes or fully characterise the mammary gland-specific posttranslational modifications of the protein and associated functionality prior to the development of fully genetically modified animals.

Recombinant protein production in rabbits required the injection of higher virus titers than in mice which is most likely due to the sheer difference in size. Extending rAAV-driven protein expression beyond rabbits to larger animals such as goats or cows will demand much larger injection volumes, even higher virus titers and further optimisation as discussed earlier. Given that rAAV are in high demand for clinical gene therapy applications, we postulate that their production costs will drop significantly in the near future. This will provide economical access to large amounts of the required high titer rAAV vectors thus opening commercial opportunities for rAAV-mediated accelerated biopharmaceutical protein production in dairy animals, such as goats.

## Methods

### Construction of rAAV plasmids

A number of expression cassettes were made for the generation of AAVs. Chicken beta actin promoter (CBA)-lacZ, CBA- green fluorescent protein (GFP) and CBA-hMBP expression cassettes and murine whey acidic protein promoter (WAP)-hMBP (Fig. 1) were separately cloned into pAM/CBL-pl-WPRE bGH expression plasmid^40^ for rAAV-mediated somatic gene transfer. A signal peptide derived from WAP and a six histidine immune-tag (6His) were included in the constructs WAP-hMBP and CBA-hMBP to direct secretion of hMBP into the milk of a lactating mammal and provide an alternative detection option for the recombinant hMBP (Fig. 1).

### rAAV preparations

Endotoxin-free plasmid preparations (Qiagen, CA) of the expression plasmids CBA-lacZ, CBA-GFP, CBA-hMBP, WAP-hMBP; the AAV1, AAV8 or AAV9 helper plasmids which express AAV2 *rep* and AAV1 *cap*, AAV8 *cap* or AAV9 *cap*, respectively, and the adenovirus helper plasmid, pFΔ6, which provides the adenoviral helper functions were prepared. The three plasmids were transfected at 1:1:1 molar ratio into HEK293 cells (seeded at a density of 1.3 × 10^5^ cells/cm^2^ the day prior to transfection) using calcium phosphate transfection. Two to three days post-transfection, the transfected cells were harvested and lysed with sodium deoxycholate at a final concentration of 0.5% in combination with repeated freeze/thaw cycles. The cell lysate was clarified using low speed centrifugation and the rAAV 1 or 9 particles purified by affinity chromatography using AVB Sepharose High Performance (GE Healthcare, NJ). Captured rAAV particles were eluted from the affinity column with 50 mM glycine buffer (pH 2.7). rAAV 8 particles were purified by iodixanol gradient as previously described^41^. Eluted rAAV particles were concentrated to a volume of 200 to 400 μl and dialysed against 2xPBS/0.001% pluronic acid using Amicon 100 k MWCO concentrators (Amicon, TX).

### rAAV characterisation

The purity of the rAAV preparations was determined following separation on SDS- PAGE gels and visualisation by Coomassie Blue (Invitrogen, CA) staining. The genomic titer of the vector preparations, the number of viral particles that contain a single-stranded AAV genome, was determined by standard quantitative real-time PCR^42^. Briefly, the isolated rAAV particles were treated with DNAseI (15 min at 25 °C) prior to viral DNA extraction from 2 μl of the purified vector preparation. After the digestion of the viral capsid with proteinase K (60 min at 65 °C), the viral DNA was amplified with the rAAV specific primers WPREfor (ggc tgt tgg gca ctg aca at) and WPRErev (ccg aag gga cgt agc aga ag) using 2× Power SYBR master mix (Applied Biosystems, CA) on a real time PCR machine (Corbett RG 6000: 3 min at 95 °C followed by 40 cycles of 10 sec at 95 °C, 15 sec at 60 °C, 20 sec at 72 °C). A standard curve of diluted plasmid equivalent to 4.6 × 10^9^ to 1 × 10^13^ vg was used to determine virus titers of rAAV preparations. Titers for the various preparations that were used in the reported experiments are summarised in Supplementary table S1.

### Transduction of mammary glands with rAAV

Animal injections were performed essentially as described by Sanchez *et al.*^14^. Briefly, one or two opposite inguinal teats of anesthetized mice or rabbits were injected with 50 μl (mice) or 600 μl (rabbits) of rAAV vector solution using a 60 to 80 μm glass micropipette (Supplementary Fig. S1). In rabbits, four to five ducts were injected whereas mice contain only one duct per teat. rAAVs (virus titers are indicated in the results section) were infused into the mammary gland one to five days prior to parturition or into glands of lactating animals. Milk letdown in mice was induced by oxytocin injections (0.3 U). The animals were milked periodically every week by gently massaging the mammary gland and collection of the milk with a glass capillary. In rabbits, induction of milk letdown proved unnecessary and was therefore omitted. At least two animals were used for all experiments. Sustainable MBP expression beyond the normal lactation periods of mice and rabbits was demonstrated in one animal each (up to eight and 19 weeks, respectively). All animal experiments were performed in compliance with existing New Zealand laws and were approved by New Zealand’s Environmental Protection Authority and the Ruakura Animal Ethics Committee.

### Recombinant protein detection assays

rAAV1-CBA-lacZ-infused mice were sacrificed two weeks after injection. Mammary glands were whole-mounted and the expression of the LacZ reporter gene was detected by an enzymatic assay for beta-galactosidase activity. The dissected mammary tissue was fixed for 2 hours in ice-cold fixative (2% paraformaldehyde, 0.002% glutaraldehyde in PBS) and then rinsed thoroughly with PBS before it was incubated in X-gal reaction buffer (0.1 M sodium phosphate buffer, ph 7.3, 2 mM MgCl_2_, 5 mM potassium ferricyanide, 5 mM potassium ferrocyanide, 0.02% NP-40 and 0.01% sodium deoxycholate) for 2 hours. The tissue was placed into fresh X-gal reaction buffer containing 1 mg/mL of X-gal solution (5-bromo-4-chloro-3-indolyl-β-D-galactoside in dimethyl sulfoxide) and incubated at 30 °C until sufficiently stained. To produce sections, stained tissue was embedded in wax, cut as 7 μm sections with a microtome, dewaxed and hydrated and mounted on polylysine-covered coverslips. Production and quantification of recombinant hMBP in milk was determined by western blot analysis using 10 μl of mouse and rabbit milk samples, that were diluted 1:100 and 1:20, respectively. Standard techniques for SDS-PAGE and protein transfer were applied and hMBP was detected either with a primary monoclonal rat anti MBP (Abcam) and a secondary horseradish peroxidase-conjugated rabbit anti rat (Sigma) antibody or a mouse anti 6His (Roche) and a secondary horseradish peroxidase-conjugated goat anti mouse (Sigma) antibody. To determine MBP protein concentrations, mouse and rabbit milk samples were separated and detected by western blot alongside known amounts of commercially-available human purified MBP (pMBP). Pixel intensities of the MBP-specific signals were measured by densitometry (GS800; BioRad) to deduct the hMBP concentration in our test samples.

All methods were carried out in accordance with the approved guidelines.

## Additional Information

**How to cite this article**: Wagner, S. *et al.* Adeno-associated-virus-mediated transduction of the mammary gland enables sustained production of recombinant proteins in milk. *Sci. Rep.* **5**, 15115; doi: 10.1038/srep15115 (2015).

## Supplementary Material

Supplementary Information
