# Supplementary material for: Adeno-associated-virus-mediated transduction of the mammary gland enables sustained production of recombinant proteins in milk
Source: Sci Rep. 2015 Oct 14;5:15115. doi: 10.1038/srep15115 (PMC4604487; doi:10.1038/srep15115)
Supplement: Supplementary Information [file srep15115-s1.pdf]

Adeno-associated-virus-mediated transduction of the mammary gland enables sustained production of recombinant proteins in milk.

Stefan Wagner, Rosemary Thresher, Ross Bland and Götz Laible

Supplementary Figure S1

a b

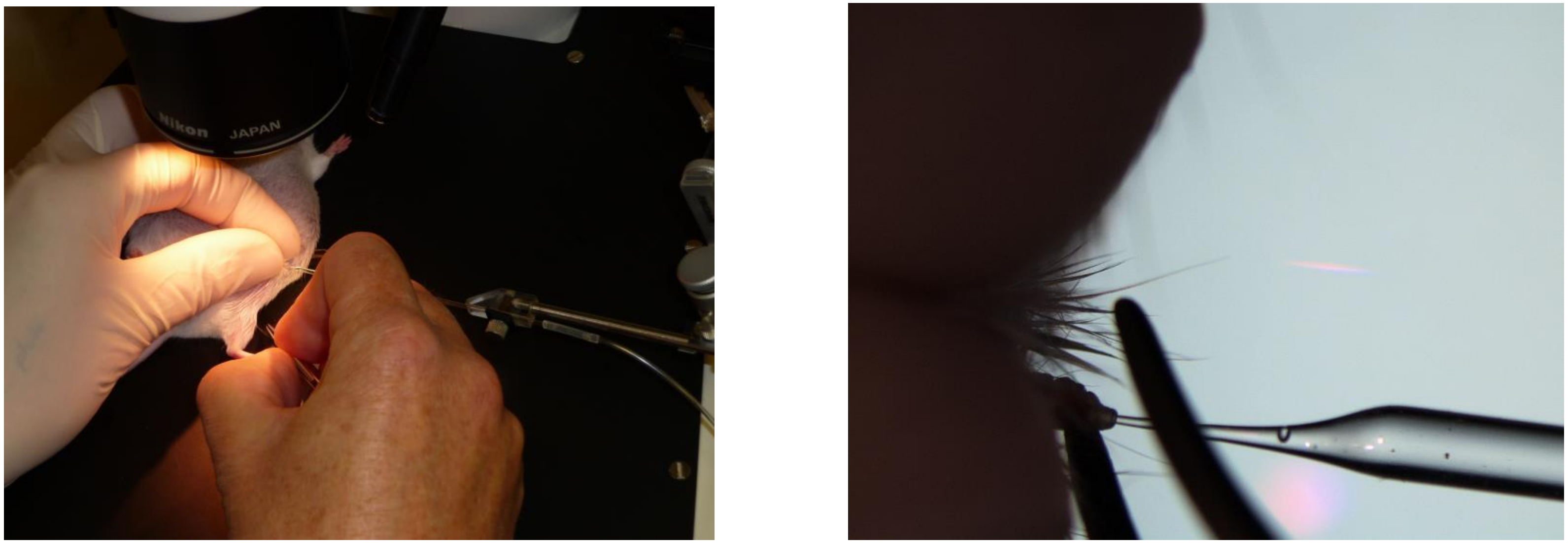

Figure S1. rAAV injection in mice. rAAV injection in the teat of day 15 pregnant mouse using a blunted glass capillary with a diameter of 60 to 80 μm. a) experimental set-up at the microscope. b) close-up of the injection with glass capillary and tweezers.

Supplementary Figure S2: original, uncropped Western blot image of Figure 7

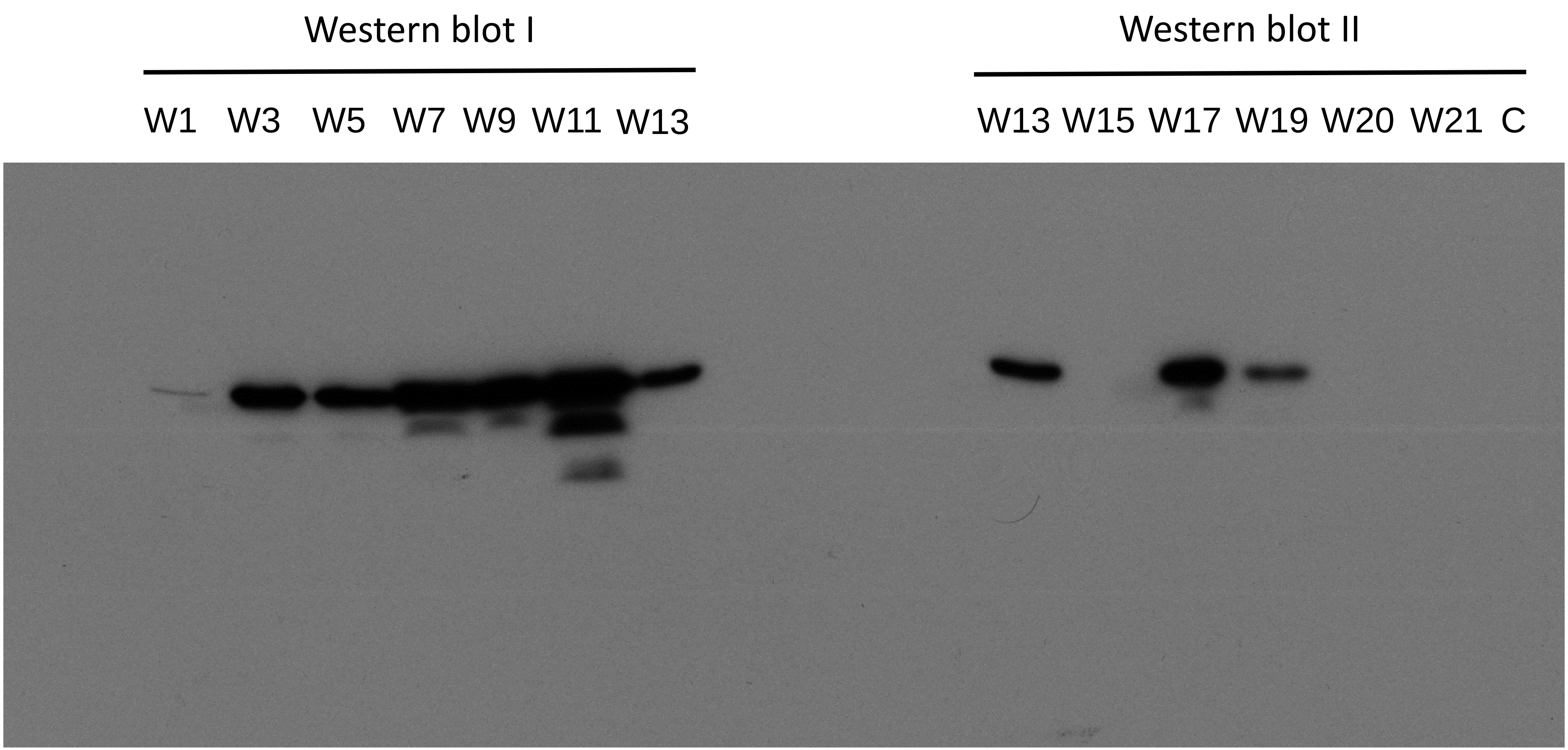

Figure S2. MBP expression in a rAAV1-CBA-MBP-injected rabbit persists over 19 weeks. Western blot with a MBP-specific antibody with milk samples of a rAAV1-CBA-MBP (3.2×10^{12} vg) injected rabbit. W1 to W21 – week 1 to week 21. C – control, milk from a non-injected gland. Gels/blots were processed in parallel.

Supplementary Table S1: Summary of viral particle titers, determined by qPCR, for all rAAV preparations that were used in different experiments as indicated.

rAAV batch	Titer [vg/ml]	Injected animal(s)/figure(s)
rAAV1 CBA-lacZ	5.3 exp 12	Mouse/Figure 2
rAAV8 CBA-EGFP	1 exp 12	Mouse/Figure 2
rAAV9 CBA-EGFP	1.75 exp 12	Mouse/Figure 2
rAAV1 WAP-MBP	2.4 exp 13	Mouse/Figure 3, 4, , 6, 8
rAAV1 CBA-MBP-1	1.4 exp 12	Mouse/Figure 3, 4, , 6, 8
rAAV1-CBA-MBP-2	1 exp 13	Mouse/Figure 6b
rAAV1 CBA-MBP-3	1.7 exp 13	Mouse/Figure 5, 8<br>Rabbit/Figure 7, 8
